# Utilisation of supplementary prenatal screening and diagnostics in Germany: cross-sectional study using data from the KUNO Kids Health Study

**DOI:** 10.1186/s12884-022-04692-1

**Published:** 2022-05-24

**Authors:** Johanna Mayer, Susanne Brandstetter, Christina Tischer, Birgit Seelbach-Göbel, Sara Fill Malfertheiner, Michael Melter, Michael Kabesch, Christian Apfelbacher, KUNO Kids Study group, Petra Arndt, Andrea Baessler, Mark Berneburg, Stephan Böse- O’Reilly, Romuald Brunner, Wolfgang Buchalla, Sara Fill Malfertheiner, Andre Franke, Sebastian Häusler, Iris Heid, Caroline Herr, Wolfgang Högler, Sebastian Kerzel, Michael Koller, Michael Leitzmann, David Rothfuß, Wolfgang Rösch, Bianca Schaub, Bernhard H. F. Weber, Stephan Weidinger, Sven Wellmann

**Affiliations:** 1 University Children’s Hospital Regensburg (KUNO), Hospital St. Hedwig of the Order of St. John, Steinmetzstr., 1-3, 93049 Regensburg, Germany; 2 Medical Sociology, Institute of Epidemiology and Preventive Medicine, University of Regensburg, Regensburg, Germany; 3 Institute for Social Medicine and Health Systems Research (ISMG), Leipzigerstr. 44, 39120 Magdeburg, Germany; 4 Research and Development Campus (WECARE), Hospital St. Hedwig of the Order of St. John, Regensburg, Germany; 5 State Institute of Health, Bavarian Health and Food Safety Authority, Bad Kissingen, Germany; 6 University Department of Obstetrics and Gynecology, Hospital St. Hedwig of the Order of St. John, University Medical Center Regensburg, Regensburg, Germany

**Keywords:** Supplementary prenatal screening and diagnostics, Andersen’s Behavioural Model of Health Services Use, Birth cohort

## Abstract

**Background:**

Appropriate health system utilisation during pregnancy is fundamental for maintaining maternal and child’s health. To study the use and determinants of supplementary prenatal screening and diagnostics in Germany this study provides comprehensive data.

**Methods:**

We obtained data from a recently established prospective German birth cohort study, the KUNO Kids Health Study. Analyses are based on Andersen’s Behavioural Model of health system use, which distinguishes between predisposing (e.g. country of birth), enabling (e.g. health insurance) and need factors (e.g. at-risk pregnancy). We examined bi- and multivariate association with the use of supplementary prenatal screening and diagnostics using logistic regression.

**Results:**

The study has a sample size of 1886 participating mothers. One fifth of the mothers investigated did not use any supplementary prenatal screening or diagnostics. Notably, the chance of using supplementary prenatal screening and diagnostics more than doubled if the pregnant woman had a private health insurance (OR 2.336; 95% CI 1.527–3.573). Higher maternal age (OR 1.038; 95% CI 1.006–1.071) and environmental tobacco smoke exposure (OR 1.465 95% CI 1.071–2.004) increased the use of supplementary prenatal screening and diagnostics. However, regarding need factors only having an at-risk-pregnancy (OR 1.688; 95% CI 1.271–2.241) showed an independent association.

**Conclusion:**

The important role of the type of health insurance and the relatively small influence of need factors was surprising. Especially with respect to equity in accessing health care, this needs further attention.

**Supplementary Information:**

The online version contains supplementary material available at 10.1186/s12884-022-04692-1.

## Background

Medical-technical progress of recent years has contributed to an improvement in prenatal care [1]. Inadequate or insufficient use of antenatal care is seen as a main risk factor for adverse pregnancy outcomes. Appropriate health system utilisation during pregnancy allows providing information about prevention programmes and to ensure that adequate therapy is initiated in case of pregnancy-specific or concurrent diseases. On the other hand, a further increase in screening programmes and additional examinations burdens health systems due to an increase in costs [2]. Hence, appropriate health system utilisation during pregnancy is important, as it can maintain maternal and child’s health [3] and can contribute to a cost-effective health care system.

In Germany the Federal Joint Committee (G-BA) defines which examinations and services are offered and the costs of which are covered by the statutory health insurance funds to all insured persons during pregnancy according to the current state of medical knowledge, considering expediency and cost-effectiveness. This includes counselling of pregnant women, examinations to detect at-risk-pregnancies, three fetal sonographies or serological examinations. Regularly visits should take place at four-week intervals, with two examinations in each of the last two months of pregnancy. If risk status arises further examinations such as additional fetal sonographies may be indicated [4]. This study focusses on the use of prenatal screening and diagnostics outside of regular care which was specified as the use of at least one medically indicated or non-medically necessary prenatal diagnostic examination: advanced 2nd trimester anatomy ultrasound, amniocenteses, first trimester screening, 3D or 4D ultrasound, cordocentesis, translucency measurement, chorionic villus sampling or non-invasive prenatal testing of maternal blood and considered dichotomized.

Prenatal genetic screening has gained importance in recent years [5]. According to a study by the Federal centre of Health education in Germany only 15% of women stated that they did not use any prenatal genetic screening [6]. A more detailed consideration reveals a decrease of invasive prenatal genetic screening in favour of an increase in non-invasive prenatal genetic screening in recent years [5]. The further development of non-invasive methods, such as the analysis of cell-free DNA from maternal blood, may lead to a further increase in prenatal genetic screening use [5, 7].

**Fig. 1:**
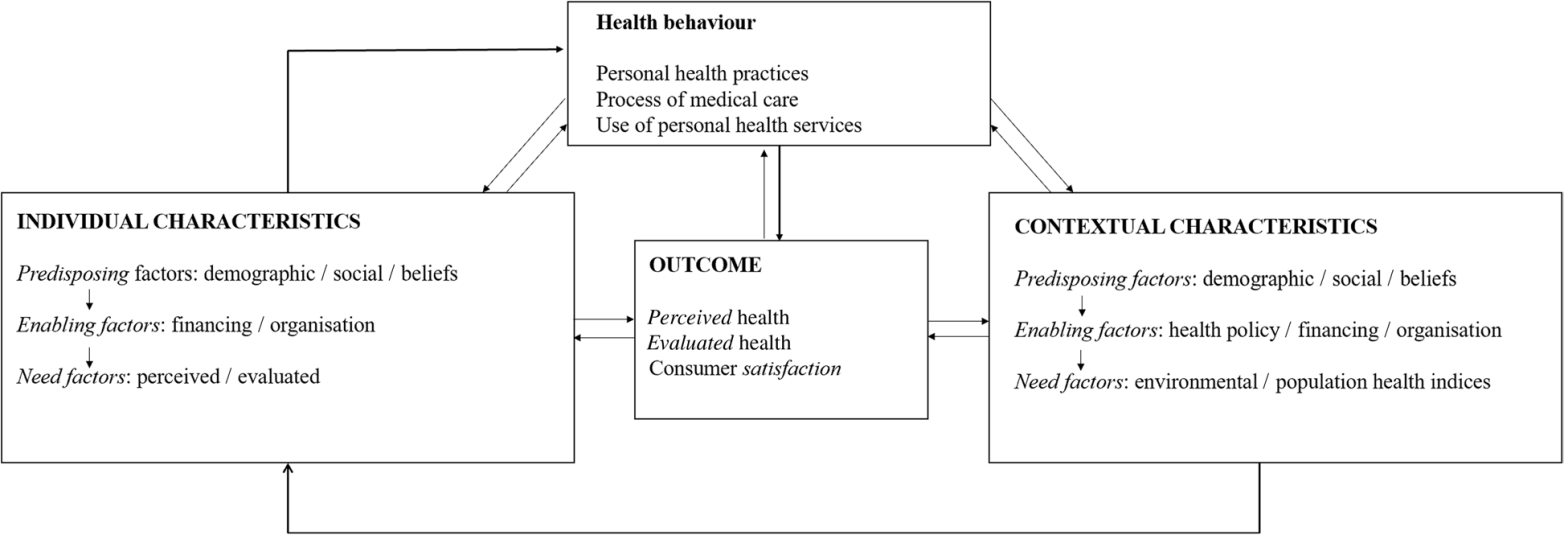
Behavioural model of health service use including contextual and individual characteristics, modified according to Andersen [10]

An established model to describe health services use is the Behavioural Model of Health Services Use developed by R.M. Andersen [8], which has been revised several times [9, 10]. The advantage of the model is, that it considers a wide range of determinants of health system use [11, 12]. Andersen defined three primary determinants of health care use: Predisposing factors including demographic characteristics such as age or ethnicity, enabling resources such as health insurance and subjectively as well as objectively surveyed need factors. Furthermore the model distinguishes between individual factors and contextual characteristics (such as accessibility of health services) [10]. For better understanding the model is illustrated in Fig. 1.

Even though this framework is frequently applied to investigate health system use [8], only a few studies have so far considered the period of pregnancy [13–17]. Among those, most researchers analysed timing or content of antenatal care [2, 15–18]. Earlier German studies solely focused on a single predictor of health care utilisation during pregnancy, such as migration [19–21] or physical activity [22]. However, to the best of our knowledge, studies using Andersen’s model to describe the use of medically indicated and non-medically necessary prenatal diagnostic examinations beyond the regular preventive examinations during pregnancy in Germany are lacking.

We addressed this research gap using data from a recently established prospective birth cohort study, the KUNO Kids health study, to provide current data on the utilisation of supplementary prenatal screening and diagnostics in Germany as well as to identify influencing factors. Analyses are based on Andersen’s Behavioural Model of Health System Use [10].

## Materials and methods

We obtained data from a prospective birth cohort study, the KUNO Kids Health Study, initiated in June 2015 at St. Hedwig hospital in Regensburg in Eastern Bavaria. The study aims to investigate a wide range of potential factors influencing various health-related outcomes in an interdisciplinary manner. Study design and procedures are described in more detail elsewhere [23].

St. Hedwig hospital is a level 1 perinatal centre with over 3000 births per year and about two thirds of the children in the region are born there [23]. The catchment area includes the city of Regensburg with 164000 inhabitants and the surrounding rural regions and is characterised by one of the lowest unemployment rates in Germany as well as rising population figures [24].

All mothers who gave birth at St. Hedwig hospital in Regensburg were asked within 48 hours after delivery for voluntary participation. Written informed consent was obtained. Criteria for exclusion were insufficient German language skills and maternal age less than 18 years. The study has been approved by the Ethics Committee of the University of Regensburg (file number: 14–101-0347).

### Data collection

Information about maternal health system utilisation during pregnancy and influencing factors was collected retrospectively through a standardised interview by study team members and self-administered questionnaires. The interview was conducted by study team members during the hospital stay after delivery. Immediately after the interview the baseline questionnaire was handed out to the mother and completed independently. Study team members rated maternal German language skills after the interview. Information about maternal age was taken directly from the electronic hospital chart.

### Predictor variables

Variables were characterised as predisposing, enabling and need factors according to the Andersen model. Table 1 provides an overview of the grouping of the predictor variables. A more detailled description of the variables assessed is provided in supplementary information section (Additional file 1).

**Table 1:** Predictor variables

Predisposing Factors	Enabling Factors	Need Factors
Maternal age	Health insurance	At-risk-pregnancy
Primiparous/multiparous	Travel time to obstetrician	Complications during pregnancy
Single parenting	Health literacy	Pre-existing illness
Country of birth other than Germany	Social support	
German language skills		
Education level		
No employment before maternity leave		
Smoking behaviour		
Physical activity in the year before pregnancy		
Unhealthy diet		

### Predisposing factors

Predisposing factors included maternal age (years), parity (primi−/multiparous), single-parenting (yes/no), country of birth (Germany/other than Germany), German language skills (excellent/lack of excellent German language skills), educational attainment (more than 10 years, 10 years, less than 10 years), employment before maternity leave (yes/no), smokers living in the household (yes/no), physical activity in the year before pregnancy (no/less than one hour per week/1–2 hours per week/ more than 2 hours per week), unhealthy diet (yes/no). Unhealthy diet was defined as fruit or vegetable consumption less than once a day.

### Enabling factors

Enabling factors considered the type of health insurance (private/statutory), traveling time to obstetrician (less than 15 min, 15 to 30 min, 30 to 60 min, more than 60 min), health literacy (see definition below) and social support (see definition below).

Health literacy is characterised as the ability to understand health related information concerning treatment options and health conditions, to know where to seek for care as well as the ability to take one’s medication correctly and being able to make appropriate health decisions [25, 26]. We assessed maternal health literacy with the health care scale of the European Health Literacy Survey (HLS-EU-Q47). Questions concerning health literacy were part of the interview. The answers (ranging from very difficult to very easy) are rated on a four-point Likert scale. The sum of the items leads to a score between 0 and 50 points, whereas a higher score level is associated with higher health literacy. Additionally, 4 groups may be performed inadequate (0–25), problematic (> 25–33), sufficient (> 33–42) and excellent health literacy (> 42–50). For the statistical calculations carried out in this paper, health literacy was analysed as a continuous variable [27].

We used the short version of the social support questionnaire (F-SozU K-14) in order to assess the level of perceived social support. The questions of the F-SozU K-14 were part of the baseline questionnaire. A total score was derived by the sum of all items (coded from 1 to 5) divided by the number of items, with higher values indicating a higher level of perceived social support [28].

### Need factors

Concerning need factors having an at-risk-pregnancy (yes/no), having hypertension or diabetes during pregnancy (yes/no), having preterm contractions, jaundice or HELLP (Hypertension, Elevated Liver enzymes and Low Platelets) (yes/no) as well as pre-existing illnesses (yes/no) was regarded. All these questions (including whether it was an at-risk-pregnancy or not) were answered by the mother in the interview. The variable at-risk-pregnancy refers to the definition of the maternity guideline catalogue, respectively the entry in the maternal routine care document (so called “Mutterpass”). It was assessed by self-report in the standardized interview and not medically verified, but as the interview was conducted by trained study team members the mother was well informed about the criteria.

### Outcome

This study focusses on supplementary prenatal screening and diagnostics which was specified as the use of at least one medically indicated or non-medically necessary prenatal diagnostic examination beyond the regular preventive examinations during pregnancy: advanced 2nd trimester anatomy ultrasound, amniocenteses, first trimester screening, 3D or 4D ultrasound, cordocentesis, translucency measurement, chorionic villus sampling or non-invasive prenatal testing of maternal blood and considered dichotomized. If any of these examinations was performed the outcome “supplementary prenatal diagnostics” was considered “yes”, if none of them was used the outcome was considered “no”. This means that the examinations covered in the analyses go beyond recommendations for routine prenatal diagnostics in case of a non-at-risk pregnancy and are independent of the medical indication. However, they are recommended and therefore covered by the statutory health insurance if medically necessary for example a 3/4D ultrasound if a hearth failure is suspected [4].

### Statistical analyses

We conducted statistical analyses using IBM SPSS statistics 24 [29]. In a first step we performed descriptive analysis to describe the study population. Associations between predictor and outcome variables were calculated using univariable logistic regression. In a second step, we performed multivariable predictive regression analyses to quantify the independent effect of each single variable. All variables with a *p*-value smaller than 0.2 in univariable analysis were included in the multivariable model. Odds ratios (OR) with 95% confidence intervals (CIs) were computed.

## Results

**Fig. 2:**
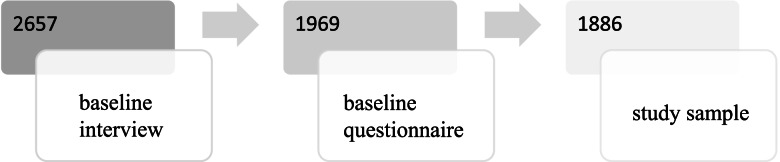
Study sample

Two thousand six hundred fifty seven infants and their families have joined the study between its start on 27th June 2015 and 28th June 2018. The study sample is defined as all mothers who participated in the interview and answered the baseline questionnaire. After excluding cases with missing values on the analytical variables, 1886 cases were left for analyses (Fig. 2).

A non-responder analysis was also conducted for a selected observation period. This showed that one third of all potential families could be included in the study. Reasons for non-participation were, for example, a stay at the intensive care unit or an outpatient birth. The entire detailed non-responder analysis can be found elsewhere [23].

Mean maternal age at delivery was 34 years, less than half of all mothers were multiparous. Twelve percent were born outside Germany and in 21% of all families, smokers were living in the household. 36% were physically inactive in the year before pregnancy and 15% had a private health insurance. Mean level of health literacy was 35.6%. A large part of the population studied shows sufficient levels of health literacy (42.7%), however 38.8% had an inadequate or problematic literacy and only 18.5% an excellent health literacy. As the clinic St. Hedwig is level one perinatal centre the number of at-risk pregnancies was relatively high (42%), as was the proportion of women having a pre-existing illness (65%). One fifth did not use any supplementary prenatal diagnostics.

Further characteristics of the study population are shown in Table 2.

**Table 2:** Characteristics of the study population

**Predisposing factors**	**Mean**	**SD**	**Min / Max**
Maternal age (years)	34.33	4.463	19 / 49
	**N**		**%**
Multiparous	847		45.2
Single parenting	44		2.4
Country of birth other than Germany	226		12.2
No excellent German language skills	94		5.7
Educational attainment			
Less than 10 years of education	26		1.4
10 years of education	716		39.3
More than 10 years of education	1082		59.3
No employment before maternity leave	207		11.2
Smokers living in the household	397		21.4
Physical activity in the year before pregnancy			
No physical activity	669		35.8
Less than 1 hour per week	204		10.9
1–2 hours per week	476		25.5
More than 2 hours per week	519		27.8
Unhealthy diet	870		47.1
**Enabling factors**			
Private health insurance	278		15
Travel time to obstetrician			
< 15 min.	635		34.3
15–30 min.	839		45.3
30–60 min.	348		18.8
> 60 min.	31		1.7
	**Mean**	**SD**	**Min / Max**
Health literacy (European Health Literacy Survey (HLS-EU-Q47)^a^	35.62	7.317	0 / 50
Social support (short version of the social support questionnaire (F-SozU K-14)^b^	4.43	0.525	1.2/ 5.0
**Need factors**	**N**		**%**
At-risk-pregnancy	786		42.6
Hypertension or diabetes during pregnancy	391		21.2
Preterm contractions, jaundice or HELLP	288		15.6
Pre-existing illness (diabetes mellitus type 1, diabetes mellitus type 2, liver disease, kidney disease, thyroid disease, hip dysplasia, cancer, coagulation disorder, cardiac arrhythmia, heart attack, heart failure, hypertension prior to the pregnancy, pyelonephritis, urological disease, other metabolic disease, ADHD, depression, anorexia, bulimia, migraine, anxiety or panic disorder, multiple sclerosis, peripheral facial nerve palsy, febrile seizure, epilepsy, single seizure, meningitis, encephalitis)	1192		64.5
**OUTCOME**	**N**		**%**
Supplementary prenatal diagnostics use	1453		79.9

*SD* Standard deviation

^a^Interview

^b^Baseline questionnaire

### Univariable analyses

In univariable analyses (Table 3), the predisposing factor maternal age indicated a higher chance of supplementary prenatal screening and diagnostics use. No employment before maternity leave was associated with a reduced chance for using supplementary prenatal diagnostics.

**Table 3:** Results from univariable logistic regression analyses

Variable	OR	Significance	95% CI
**Predisposing factors**			
Multiparous	0.911	0.426	[0.724; 1.147]
**Maternal age**	**1.073**	< 0.001**	**[1.045; 1.102]**
Single parenting	1.205	0.658	[0.529; 2.746]
Country of birth other than Germany	0.995	0.976	[0.696; 1.421]
No excellent German language skills	1.308	0.371	[0.727; 2.352]
Education level (compared to < 10 years)			
10 years	1.167	0.748	[0.455; 2.991]
> 10 years	1.430	0.454	[0.560; 3.649]
**No employment before maternity leave**	**0.691**	0.034*	**[0.491; 0.973]**
Smokers living in the household	1.316	0.070	[0.977; 1.773]
Physical activity in the year before pregnancy (compared to no physical activity)			
< 1 hour/week	1.130	0.556	[0.753; 1.695]
1–2 hours/week	1.007	0.966	[0.748; 1.355]
> 2 hours/week	1.003	0.984	[0.750; 1.341]
Unhealthy diet	0.966	0.772	[0.767; 1.218]
**Enabling factors**			
**Private health insurance**	**2.498**	< 0.001**	**[1.659; 3.761]**
Travel time to obstetrician (compared to < 15 min.)			
15–30 min.	1.073	0.595	[0.827; 1.394]
30–60 min.	1.194	0.309	[0.849; 1.679]
> 60 min.	1.634	0.370	[0.558. 4.780]
Health literacy	1.013	0.109	[0.997; 1.029]
Social support	0.882	0.288	[0.700; 1.112]
**Need factors**			
**At-risk-pregnancy**	**1.944**	< 0.001**	**[1.519; 2.489]**
Hypertension or diabetes	1.025	0.869	[0.768; 1.368]
Preterm contractions, jaundice or HELLP	1.034	0.839	[0.750; 1.425]
**Pre-existing illness**	**1.313**	0.025*	**[1.036; 1.665]**

*OR* Odds Ratio, *95% CI* 95% confidence interval, *P-Value < 0.05, ** *P*-Value < 0.01

Regarding enabling factors having a private health insurance showed a positive association with supplementary prenatal screening and diagnostics use.

Concerning need factors only a reported at-risk-pregnancy and having a pre-existing illness indicated a higher chance for supplementary prenatal screening and diagnostics use in the univariable model.

### Multivariable analyses

The chance of using supplementary prenatal screening and diagnostics (Table 4) increased significantly with increasing maternal age and was also significantly increased when smokers were living in the household. However, being unemployed before maternity leave did not remain significant in the multivariable model. For enabling characteristics, the chance of using supplementary prenatal screening and diagnostics more than doubled if the mothers had a private health insurance. Similarly, a reported at-risk-pregnancy significantly increased the chance of supplementary prenatal screening and diagnostics use with respect to need characteristics. Having a pre-existing illness did not remain significant in the multivariable model (Table 4).

**Table 4:** Results from multivariable logistic regression analyses

Variable	OR	Significance	95% CI
**Predisposing factors**			
Maternal age	1.038	0.018*	[1.006; 1.071]
Smokers living in the household	1.465	0.017*	[1.071; 2.004]
**Enabling factors**			
Private health insurance	2.336	< 0.001**	[1.527; 3.573]
**Need factors**			
At-risk-pregnancy	1.688	< 0.001**	[1.271; 2.241]

*OR* Odds Ratio, *95% CI* 95% confidence interval, *P-Value < 0.05, ** *P*-Value < 0.01

## Discussion

The present study assessed the amount and determinants of supplementary prenatal screening and diagnostics use. Higher maternal age and environmental tobacco smoke exposure increased the chance for the use of supplementary prenatal diagnostics. Notably having a private health insurance showed a strong association with higher odds of supplementary prenatal diagnostics. With respect to this, the chance of using supplementary prenatal screening and diagnostics more than doubled if the mother had a private health insurance. However, regarding need factors only having an at-risk-pregnancy was independently associated with supplementary prenatal screening and diagnostics use.

To the best of our knowledge, this is the first study using Andersen’s model to describe supplementary prenatal screening and diagnostics use in Germany. Earlier studies mainly focused on timing and content of antenatal care [2, 14–18]. Further, most studies regarding prenatal diagnostics were set outside of Germany [30, 31] or did not apply Andersen’s model [5]. Therefore, comparability is limited. We set out to address this research gap to provide precise data about the amount and influencing factors of supplementary prenatal screening and diagnostics use in Germany.

### Predisposing factors

Higher maternal age is associated with at-risk pregnancies which may lead to an increased use of supplementary prenatal diagnostics, especially as further prenatal diagnostics is covered by health insurance due to risk status [32]. Our findings identifying maternal age as predictor for the use of supplementary prenatal screening and diagnostics are in line with a recent study describing maternal age as the strongest predictor for undergoing invasive genetic testing, respectively the use of prenatal diagnostics in general [30].

Our results indicate a higher chance of using supplementary prenatal screening and diagnostics if smokers are living in the household. This may be partly explained by findings reporting a higher utilisation of medical services among smokers in general [33, 34]. Even though the data of these studies did not permit an analysis of the causes, higher morbidity rates due to smoking [33] as well as a less health conscious behaviour [34] were discussed. However, our results are in contrast to previous studies reporting smoking as risk factor for an inadequate use of antenatal care [14, 15] or lower degrees of undergoing combined ultrasound and biochemical test [30].

A possible explanation for our diverging results could be that most studies examined the association between smoking and health service utilisation. In our analyses, however, “smokers living in the household” was used as independent variable. Thus, especially in those cases where only the father is a smoker, the mother as a non-smoker could be particularly aware of the risks for the child and therefore have higher utilisation rates of supplementary prenatal diagnostic programmes. Additionally, it has to be taken into account that the awareness regarding health risks of smoking during pregnancy has increased in recent years. With respect to this, the increased use of additional prenatal diagnostics could also be seen as success of these prevention programmes.

### Enabling factors

To better understand the results concerning health insurance, it is important to know, that in Germany, both privately and statutorily insured persons are usually treated in the same clinics. However, the medical fee differs depending on the insurance [35]. Some private health insurances also cover services that go beyond those covered by the statutory health insurance [36].

The role of private health insurance we identified is in line with previous research that reported social differences in health system utilisation in Germany [32, 37]. A range of studies identified higher utilisation rates of preventive health care for higher socio-economic status groups, better educated or people with a private health insurance, whereas socially disadvantaged show increased hospitalisation rates [38–41]. These findings are supported by our data. There are several possible explanations. First, differences may be caused due to different information status about health care services or health in general. This may contribute especially to the lack in the use of preventive care [42]. A further explanation may be Andersen’s hypothesis that the use of elective health services is mainly explained by enabling resources whereas for the use of hospitals need factors are more important, as they are mainly consulted due to more serious problems [10, 43].

The above-mentioned findings are according to our analyses concerning educational attainment, which revealed a higher chance for the use of supplementary prenatal screening and diagnostics with a higher educational attainment. However, the association was not significant. This is in line with findings from the Robert Koch Institute which did not support an association between education and health care use, but strong differences according to social conditions [44].

### Need factors

Most studies addressing health system utilisation reported need factors as strong factors involved [11, 13]. However, regarding supplementary prenatal screening and diagnostics only the variable “having an at-risk-pregnancy” showed an independent association, whereas no significant association between the other need factors and the use of supplementary prenatal screening and diagnostics was found. The positive association between an at-risk-pregnancy and supplementary prenatal screening and diagnostics use is consistent to an increase in the number of prenatal visits when risk status arose in a study of Feijen-de-Jong et al. [14]. The relatively small association with need factors in general and the important role of the type of health insurance was surprising and worrisome as it indicates social disparities in supplementary prenatal screening and diagnostics use, however it contributes to the above mentioned hypothesis by Andersen et al. [43, 45].

### Frequency of utilisation of supplementary prenatal diagnostics

Regarding the frequency of use of supplementary prenatal screening and diagnostics comparisons are limited as official statistics are lacking and most studies analysed utilisation rates for special examinations such as translucency measurement and not the total amount of supplementary prenatal screening and diagnostics use [5]. For example, the 2015 Health Monitor reported around 50% uptake of a 3/4D ultrasound [1].

### Strengths and limitations

As the St. Hedwig hospital is a level one perinatal centre it covers a major part of births in the region of Eastern Bavaria. However, there is a relatively high proportion of women with at-risk pregnancies [23] which could lead to higher health care utilisation rates during pregnancy. On the other hand, the prevalence of at-risk-pregnancies in the study sample is 42%, which does not differ considerably from the Bavarian average of 36% [46]. A further limitation is that at-risk-pregnancy was assessed by self-report and not verified by a medical diagnosis. However, it was assessed in the standardised interview by trained study team members after precisely informing the mother about the criteria referring to the maternity guideline catalogue.

The use of self-report data on the utilisation of supplementary prenatal screening and diagnostics is a limitation of our study. Bias due to memory effects cannot be fully excluded. Despite, as these examinations require information and consent of the mother before being conducted it can be supposed that they are well remembered.

Due to language barriers migrants are often underrepresented in population based research [23, 47]. This is also a potential limitation of our study, as we excluded participants with insufficient German language skills to give informed consent. However, the percentage of mothers having another country of birth than Germany is approximately consistent with official statistics for women in German population and for the region [37].

There is an underrepresentation of the lowest education group [48]. This may be an explanation why educational level did not show a significant association to supplementary prenatal screening and diagnostics use. Nevertheless, also findings from the Robert Koch Institute did not support an association between education and supplementary prenatal screening and diagnostics use [44]. Furthermore due to overrepresentation of well-educated women the study population may show higher health literacy levels as previous findings for a representative sample of the German population [27]. However, in general, it can be said that women who would otherwise have been unattainable, could be included to the study due to the special efforts in recruitment such as presenting the study to each mother by study team members.

## Conclusion

The study provides comprehensive data from a large sample of mothers on the utilisation of health care during pregnancy, as well as potentially influencing factors. Especially the strong influence of the type of health insurance as well as the relatively small importance of need factors have to be taken into account and considered when discussing equity in accessing health care. The present study focusses on the use of prenatal diagnostics outside of regular care without reference to the medical indication. However, the insights gained may be used as basis to establish further research to identify ways to use health system in a needs-based manner by presenting intervention options, such as reducing differences between statutory and private health insurance in order to prevent missing a diagnosis due to not using an important examination, or on the other hand not causing unnecessary concern due to the use of irrelevant examinations. Therefore, it is important to provide the information mothers need to discriminate about the adequacy of screening.

## Supplementary Information

**Additional file 1.**

## Data Availability

The datasets used and analysed for this paper are available from the corresponding author on reasonable request.

## Abbreviations

HELLP: Hypertension, Elevated Liver enzymes and Low Platelets
HL: Health literacy
KUNO: Kinder Uniklinik Ostbayern (children’s university hospital for the region of Eastern Bavaria)
