# Supplementary material for: Utilisation of supplementary prenatal screening and diagnostics in Germany: cross-sectional study using data from the KUNO Kids Health Study
Source: BMC Pregnancy Childbirth. 2022 May 24;22:436. doi: 10.1186/s12884-022-04692-1 (PMC9131677; doi:10.1186/s12884-022-04692-1)
Supplement: Supplementary file 1 — Additional file 1. [file 12884_2022_4692_MOESM1_ESM.docx]

Additional file: description of the variables assessed during the interview or baseline questionnaire

| **Predisposing factors** | **Response option** | **Data source** | **Operationalisation of constructs** |
| --- | --- | --- | --- |
| **Of the** |  |  |  |
| maternal age | years | electronic hospital chart |  |
| parity | primi-/multiparous | baseline interview |  |
| single-parenting | yes/no | baseline interview |  |
| country of birth | Germany/other than Germany | baseline interview |  |
| German language skills | excellent/lack of excellent German language skills | rated by study team members after the baseline interview |  |
| educational attainment | more than 10 years, 10 years, less than 10 years | baseline interview |  |
| employment before maternity leave | yes/no | baseline interview |  |
| smokers living in the household | yes/no | baseline interview |  |
| physical activity in the year before pregnancy | no/less than one hour per week/1-2 hours per week/ more than 2 hours per week | baseline interview |  |
| unhealthy diet | yes/no | baseline interview | The amount of fruit and vegetable consumption during pregnancy was assessed. Unhealthy diet was defined as fruit or vegetable consumption less than once a day. |
| **Enabling factors** |  |  |  |
|  |  |  |  |
| type of health insurance | private/statutory | baseline interview |  |
| traveling time to obstetrician | less than 15 minutes, 15 to 30 minutes, 30 to 60 minutes, more than 60 minutes | baseline questionnaire |  |
| health literacy | score between 0 and 50 points | baseline interview | health care scale of the European Health Literacy Survey (HLS-EU-Q47) |
| social support | score between 1 and 5 points | baseline questionnaire | short version of the social support questionnaire (F-SozU K-14) |
| **Need factors** |  |  |  |
|  |  |  |  |
| at-risk-pregnancy | yes/no | baseline interview |  |
| having hypertension or diabetes during pregnancy | yes/no | baseline interview |  |
| having preterm contractions, jaundice or HELLP (Hypertension, Elevated Liver enzymes and Low Platelets) | yes/no | baseline interview |  |
| pre-existing illnesses | yes/no | baseline questionnaire |  |
